# FAIL-T (AFP, AST, tumor sIze, ALT, and Tumor number): a model to predict intermediate-stage HCC patients who are not good candidates for TACE

**DOI:** 10.3389/fmed.2023.1077842

**Published:** 2023-05-02

**Authors:** Apichat Kaewdech, Pimsiri Sripongpun, Suraphon Assawasuwannakit, Panu Wetwittayakhlang, Sawangpong Jandee, Naichaya Chamroonkul, Teerha Piratvisuth

**Affiliations:** ^1^Gastroenterology and Hepatology Unit, Division of Internal Medicine, Faculty of Medicine, Prince of Songkla University, Songkhla, Thailand; ^2^Department of Medicine, Panyananthaphikkhu Chonprathan Medical Center, Srinakharinwirot University, Nonthaburi, Thailand; ^3^NKC Institute of Gastroenterology and Hepatology, Songklanagarind Hospital, Prince of Songkla University, Songkhla, Thailand

**Keywords:** hepatocellular carcinoma (HCC), prognostic score, survival, transarterial chemoembolization (TACE), predictor, intermediate stage

## Abstract

**Background:**

Patients with un-resectable hepatocellular carcinoma (HCC) treated with transarterial chemoembolization (TACE) are a diverse group with varying overall survival (OS). Despite the availability of several scoring systems for predicting OS, one of the unsolved problems is identifying patients who might not benefit from TACE. We aim to develop and validate a model for identifying HCC patients who would survive <6 months after their first TACE.

**Methods:**

Patients with un-resectable HCC, BCLC stage 0-B, who received TACE as their first and only treatment between 2007 and 2020 were included in this study. Before the first TACE, demographic data, laboratory data, and tumor characteristics were obtained. Eligible patients were randomly allocated in a 2:1 ratio to training and validation sets. The former was used for model development using stepwise multivariate logistic regression, and the model was validated in the latter set.

**Results:**

A total of 317 patients were included in the study (210 for the training set and 107 for the validation set). The baseline characteristics of the two sets were comparable. The final model (FAIL-T) included AFP, AST, tumor sIze, ALT, and Tumor number. The FAIL-T model yielded AUROCs of 0.855 and 0.806 for predicting 6-month mortality after TACE in the training and validation sets, respectively, while the “six-and-twelve” score showed AUROCs of 0.751 (*P* < 0.001) in the training set and 0.729 (*P* = 0.099) in the validation sets for the same purpose.

**Conclusion:**

The final model is useful for predicting 6-month mortality in naive HCC patients undergoing TACE. HCC patients with high FAIL-T scores may not benefit from TACE, and other treatment options, if available, should be considered.

## 1. Introduction

Liver cancer is the sixth most common cancer worldwide and the third leading cause of cancer-related death (1). In 2020, there were approximately 905,000 new cases and more than 800,000 deaths (1). Hepatocellular carcinoma (HCC) accounts for > 90% of all liver cancers (2). Notably, a prediction model predicts that new HCC cases will increase by 35% by 2030 (3). The highest HCC prevalence is observed in Asian countries (72.5%), followed by Europe (9.7%) and Africa (7.8%) (1).

Treatments are stratified according to the Barcelona Clinic Liver Cancer (BCLC) staging system, which includes tumor burden, liver function, and patient performance status (PS) (4). Transarterial chemoembolization (TACE) is the main treatment modality for patients with HCC BCLC stage B (intermediate stage) and un-resectable HCC BCLC stage A (5, 6). TACE has been shown to improve survival in HCC patients in several studies (7–9).

Patients with intermediate-stage HCC and un-resectable HCC BCLC stage A represent a diverse group with a median overall survival (OS) of 13 to 32.9 months (10–17). Therefore, there have been sub-classifications in this BCLC stage to better categorize patients and guide treatment strategies, such as Bolondi's sub-classification (18), the Kinki criteria (19), and recently, the 2022 updated BCLC system (4). According to the latest BCLC system, intermediate-stage HCC can be divided into three subgroups (4). Patients with diffuse, infiltrative, or bilobar involvement are unlikely to benefit from TACE, and systemic therapy should be considered. However, there are no definite criteria for intermediate-stage HCC patients who should skip TACE and proceed to systemic treatment as their first-line option.

Therefore, this study aimed to develop a prognostic model to predict treatment-naive patients who are not good candidates for TACE. This model may be useful for guiding treatment decisions in patients with treatment-naive, unresectable HCC BCLC A and B. We hypothesized that patients who survived <6 months after their first TACE should not have TACE as their first-line treatment. Therefore, the factors associated with 6-month mortality were explored in this study.

## 2. Materials and methods

### 2.1. Patients and study design

This retrospective cohort study was conducted at a super-tertiary university hospital that serves as a referral center for patients from southern Thailand. Medical records from an electronic hospital database (Health Information System, HIS) were screened for patients diagnosed with HCC (ICD-10 code C229, malignant neoplasm of the liver, unspecified and code C220, malignant neoplasm of liver cell carcinoma) between 1 January 2007 and 31 December 2020. According to the American Association for the Study of Liver Disease (AASLD) (20), the European Association for the Study of Liver (EASL) (5), or the Asian Pacific Association for the Study of the Liver (APASL) guidelines (21), HCC was diagnosed using imaging or histology. The inclusion criteria were as follows: age ≥18 years, first diagnosis of unresectable HCC with BCLC stage 0, A or B, Child–Pugh score A5-B7, and receiving TACE as their monotherapy. Exclusion criteria included HCC in conjunction with other active malignancies, a history of spontaneous tumor rupture, co-treatment with any systemic, locoregional therapies or liver transplantation, liver decompensation prior to TACE, and a lack of baseline imaging information. Baseline clinical characteristics, such as age at diagnosis, sex, PS, etiology of chronic liver disease, Child–Pugh score, BCLC stage, and baseline alpha-fetoprotein (AFP) level, were collected on the day of TACE or no longer than 48 hours before their first TACE. HCC diagnosis and tumor burden, including tumor size and number, were determined by abdominal radiologists, as reported in the HIS. According to international recommendations, antiviral therapy was initiated in all patients with hepatitis B infection prior to their first TACE (22–24). According to the country's reimbursement policy, for patients with chronic hepatitis C infection, direct-acting antivirals were not administered during the course of HCC treatment, but were considered after HCC remission of at least 6 months.

Overall survival was defined as the time interval between the date of the first TACE and death from any cause. Patient status at the censor date of the study (23 July 2021) was defined as alive or dead using data from the Thailand Civil Registration Database.

In this study, we acquired data solely retrospectively. Therefore, the informed consent was not required. The protocol for this study was approved by the Faculty of Medicine's Institutional Review Board and Ethics Committee, Prince of Songkla University, Thailand (REC 62-302-14-1). This study was conducted in compliance with the ethical guidelines of the 1975 Declaration of Helsinki.

### 2.2. Treatment procedures

All eligible patients underwent a selective conventional TACE procedure as previously described (16). Briefly, the procedure was performed by two interventional radiologists with at least 5 years of experience. Angiography and catheterization were advanced very selectively through the subsegmental hepatic artery that fed the tumor. A mixture of lipiodol and a chemotherapeutic agent was infused and then embolized with gelatin sponge particles. TACE was scheduled on demand based on the viability of HCC post-treatment at an interval of 4–8 weeks.

### 2.3. Statistical analyses

Continuous variables such as age, tumor size, liver biochemistry results, platelets, international normalized ratio (INR), and AFP were expressed as mean (± standard deviation [SD]) or median (interquartile range [IQR]), as appropriate. Categorical variables were expressed as numbers and percentages, such as sex, cirrhosis status, etiology, Child–Pugh score, HCC stage, and albumin–bilirubin (ALBI) score classification. All patients were randomly categorized to a “training set” and “validation set” in a 2:1 ratio, respectively. For comparisons between two groups of patients, the χ^2^ test or, when appropriate, Fisher's exact test was used to compare categorical variables. Wilcoxon rank sum test or *t*-test was used to compare continuous variables according to the distribution of the data.

The training set was used for the model development. Univariable logistic regression analysis was performed to determine the variables associated with 6-month mortality. Subsequently, potential factors (*P* < 0.1) from univariable analyses and variables known to be associated with HCC survival were selected for a multivariable logistic regression analysis (25). The stepwise selection was then applied to select the independent variables to create the model. Based on the Asia-Pacific study of sorafenib in patients with advanced HCC (26), we chose 6-month mortality as a threshold for patients who are not good candidates for TACE. Asian patients with BCLC C HCC who received sorafenib treatment had a median survival of 6.5 months. We hypothesized that if patients in intermediate-stage HCC (BCLC B), the earlier stage compared to BCLC C, survived <6 months after their TACE, it indicated that the treatment did not benefit them.

Survival curves were estimated using the Kaplan–Meier method, and the log-rank test was used to compare them. The recently developed model was then validated and compared to the “six-and-twelve” scoring system. Confirmatory analysis was performed to evaluate the discrimination ability of the scoring systems by estimating the AUROC for a 6-month death prediction. The sensitivity, specificity, and positive (PPV) and negative (NPV) predictive values were calculated at the given cutoffs.

All statistical analyses were performed using R software, version 4.1.0 (R Foundation, Austria). A two-sided *P*-value of <0.05 was considered statistically significant.

## 3. Results

### 3.1. Baseline clinical characteristics

Among the 752 patients who underwent TACE during the study period, 435 were excluded (51 due to the history of decompensation prior to their first TACE, 91 due to vascular involvement or extrahepatic spreading, 40 previously treated with hepatic resection, 277 previously or subsequently treated with ablation therapy, 18 received additional systemic treatment, 48 due to the lack of baseline imaging data for review, and three due to the lack of survival data). Finally, a total of 317 treatment-naive patients with unresectable HCC BCLC stage A or B who underwent their first TACE were included. The eligible patients were randomly divided at a 2:1 ratio into two groups: training (*n* = 210) and validation (*n* = 107). Table 1 shows that the baseline characteristics of the two groups were comparable. In the entire cohort, the mean age was 61.3 years, and nearly two-thirds were men. The most common etiology of HCC was hepatitis B infection (48.9%), followed by hepatitis C infection (19.6%) and alcohol consumption (12.3%). Most patients had Child–Pugh class A (81.4%). The median tumor size and number were 3.8 cm and single lesion, respectively. More than half of the patients were classified as BCLC stage A, followed by BCLC stage B (34.7%). The majority of patients were categorized as having ALBI grade 2 (68.5%). Except for mild thrombocytopenia, all baseline laboratory data indicated that liver function was preserved, as shown in Table 1. The median AFP level was 28.7 (IQR 8.7–320) ng/mL in 23.7% of the patients, an AFP level of 400 ng/mL or higher was detected. The characteristics of patients who were alive or died 6 months after their first TACE are shown in Table 2.

**Table 1:** Baseline demographics and clinical characteristics of the patients in the training set (*n* = 210) and the validation set (*n* = 107).

**Baseline characteristics**	**Total (*n* = 317)**	**Training set (*n*= 210)**	**Validation set (*n* = 107)**	***P*-value**
Age, years, mean (SD)	61.3 (10.6)	60.8 (10.8)	62.1 (10.1)	0.329
Male sex, no. (%)	229 (72.2)	148 (70.5)	81 (75.7)	0.396
Cirrhosis, no. (%)	296 (93.4)	197 (93.8)	99 (92.5)	0.844
Etiology, no. (%)				0.603
HBV	155 (48.9)	101 (48.1)	54 (50.5)	
HCV	62 (19.6)	37 (17.6)	25 (23.4)	
Alcohol	39 (12.3)	29 (13.8)	10 (9.3)	
NAFLD	29 (9.1)	22 (10.5)	7 (6.5)	
Cryptogenic	29 (9.1)	19 (9.0)	10 (9.3)	
AIH	3 (0.9)	2 (1.0)	1 (0.9)	
Child-Pugh score, no. (%)				0.369
A5	143 (45.1)	89 (42.4)	54 (50.5)	
A6	115 (36.3)	81 (38.6)	34 (31.8)	
B7	59 (18.6)	40 (19.0)	19 (17.8)	
**Tumor characteristics**				
Largest tumor size, cm, median (IQR)	3.8 (2.4, 7.5)	4 (2.7, 8.1)	3.8 (2.1, 6.8)	0.115
Largest tumor size group, cm, no. (%)				0.220
≤3	117 (36.9)	69 (32.9)	48 (44.9)	
>3–7	116 (36.6)	82 (39.0)	34 (31.8)	
>7–10	38 (12.0)	27 (12.9)	11 (10.3)	
>10	46 (14.5)	32 (15.2)	14 (13.1)	
Tumor number, no., median (IQR)	1 (1, 3)	1 (1, 3)	1 (1, 2)	0.261
Tumor number group, no. (%)				0.783
≤3	285 (89.9)	190 (90.5)	95 (88.8)	
>3	32 (10.1)	20 (9.5)	12 (11.2)	
BCLC stage^†^, no. (%)				0.041
0	38 (12.0)	23 (11.0)	15 (14.0)	
A	169 (53.3)	104 (49.5)	65 (60.7)	
B	110 (34.7)	83 (39.5)	27 (25.2)	
ALBI grade, no. (%)				0.436
1	22 (6.9)	12 (5.7)	10 (9.3)	
2	217 (68.5)	144 (68.6)	73 (68.2)	
3	78 (24.6)	54 (25.7)	24 (22.4)	
**Laboratory results**				
TB, mg/dL, median (IQR)	0.8 (0.6, 1.3)	0.8 (0.6, 1.2)	0.8 (0.5, 1.3)	0.968
AST, U/L, median (IQR)	56 (39, 86)	57 (40, 85.8)	55 (39, 89)	0.692
ALT, U/L, median (IQR)	37 (24, 54)	37 (24, 56)	35 (23.5, 51)	0.573
Albumin, g/dL, mean (SD)	3.5 (0.5)	3.5 (0.5)	3.6 (0.5)	0.020
Platelets, 10^9^/L, median (IQR)	122 (75, 201)	122 (74, 202.5)	117 (77.5, 198.5)	0.825
INR, median (IQR)	1.2 (1.1, 1.3)	1.2 (1.1, 1.3)	1.2 (1.1, 1.3)	0.299
AFP, ng/mL, median (IQR)	28.7 (8.7, 320)	32.3 (9, 324.1)	22.8 (8, 289.6)	0.529
AFP group, ng/mL, no. (%)				1.000
<400	242 (76.3)	160 (76.2)	82 (76.6)	
≥400	75 (23.7)	50 (23.8)	25 (23.4)	
TACE sessions	2 (1, 3)	2 (1, 3)	2 (1, 4)	0.900

ABLI, albumin-bilirubin; AFP, alpha-fetoprotein; AIH, autoimmune hepatitis; ALT, alanine aminotransferase; AST, aspartate aminotransferase; BCLC, Barcelona Clinic Liver Cancer; HBV, hepatitis B virus; HCV, hepatitis C virus; INR, international normalized ratio; IQR, interquartile range; NAFLD, nonalcoholic fatty liver disease; TACE, transarterial chemoembolization; TB, total bilirubin.

Means with standard deviations and medians with IQR are shown for quantitative variables, whereas counts with proportions are shown for categorical variables. Data are expressed as medians with interquartile ranges except where noted. ^†^ With PS0, reserved liver function, and absence of metastasis or macrovascular invasion, BCLC staging considers a single tumor>2 cm as stage A.

**Table 2:** Baseline characteristics of patients in the training set stratified by status at six-month after 1^st^ TACE.

**Baseline characteristics**	**Alive (*n* = 168)**	**Death within 6-month (*n* = 42)**	***P*-value**
Age, years, mean (SD)	61.1 (11.0)	59.9 (10.4)	0.523
Male sex, no. (%)	118 (70.2)	30 (71.4)	1.000
Cirrhosis, no. (%)	160 (95.2)	37 (88.1)	0.142
Etiology, no. (%)			0.417
HBV	78 (46.4)	23 (54.8)	
HCV	33 (19.6)	4 (9.5)	
Alcohol	21 (12.5)	8 (19.0)	
NAFLD	17 (10.1)	5 (11.9)	
Cryptogenic	17 (10.1)	2 (4.8)	
AIH	2 (1.2)	0 (0)	
Child-Pugh score, no. (%)			0.195
A5	76 (45.2)	13 (31.0)	
A6	63 (37.5)	18 (42.9)	
B7	29 (17.3)	11 (26.2)	
**Tumor characteristics**			
Largest tumor size, cm, median (IQR)	3.6 (2.5, 6.1)	7.6 (4.8, 13.7)	<0.001
Largest tumor size group, cm, no. (%)			<0.001
≤3	65 (38.7)	4 (9.5)	
>3–7	66 (39.3)	16 (38.1)	
>7–10	21 (12.5)	6 (14.3)	
>10	16 (9.5)	16 (38.1)	
Tumor number, no., median (IQR)	1 (1, 3)	2 (1, 3)	0.399
Tumor number group, no. (%)			0.560
≤3	153 (91.1)	37 (88.1)	
>3	15 (8.9)	5 (11.9)	
ALBI grade, no. (%)			0.039
1	12 (7.1)	0 (0)	
2	118 (70.2)	26 (61.9)	
3	38 (22.6)	16 (38.1)	
**Laboratory results**			
TB, mg/dL, median (IQR)	0.8 (0.5, 1.2)	0.9 (0.7, 1.6)	0.005
AST, U/L, median (IQR)	52 (37, 77)	89.5 (62.5, 126.8)	<0.001
ALT, U/L, median (IQR)	36 (23.8, 50.5)	48.5 (32, 69.5)	0.007
Albumin, g/dL, mean (SD)	3.5 (0.5)	3.3 (0.4)	0.023
Platelets, 10^9^/L, median (IQR)	110.5 (69, 174.2)	170 (130, 282.8)	<0.001
INR, median (IQR)	1.2 (1.1, 1.4)	1.2 (1.2, 1.3)	0.731
AFP, ng/mL, median (IQR)	23.5 (7.8, 196.5)	624.2 (33.7, 28155.8)	<0.001
AFP group, ng/mL, no. (%)			<0.001
<400	139 (82.7)	21 (50.0)	
≥400	29 (17.3)	21 (50.0)	
TACE sessions, median (IQR)	2 (1, 4)	1 (1, 2)	<0.001

ABLI, albumin-bilirubin; AFP, alpha-fetoprotein; AIH, autoimmune hepatitis; ALT, alanine aminotransferase; AST, aspartate aminotransferase; BCLC, Barcelona Clinic Liver Cancer; HBV, hepatitis B virus; HCV, hepatitis C virus; INR, international normalized ratio; IQR, interquartile range; NAFLD, nonalcoholic fatty liver disease; TACE, transarterial chemoembolization; TB, total bilirubin.

Means with standard deviations and medians with IQR are shown for quantitative variables, whereas counts with proportions are shown for categorical variables. Data are expressed as medians with interquartile ranges except where noted.

### 3.2. Univariate and multivariate analyses' prognostic factors for a 6-month death

As mentioned earlier, the data from the training set of patients were used to evaluate the variables associated with death within 6 months and develop the scoring system. Table 3 shows the univariate analysis results for the variables associated with 6-month death. Tumor size (of the largest one if multiple masses were detected), cirrhosis, Child–Pugh stage, aspartate aminotransferase (AST), alanine aminotransferase (ALT), albumin, platelets, and AFP groups had a *P*-value of <0.1 in univariable analyses and were then entered into the multivariable analysis. Tumor number, despite having a *P*-value of >0.1 in a univariable analysis, was included in the multivariable analysis because it has been shown to be significantly associated with OS in HCC patients in previous studies (17, 27). From the multivariable logistic regression analysis, the stepwise selection was used to select the variables included in the final model. The results of the multivariable analysis with stepwise regression are shown in Table 3.

**Table 3:** Univariate and multivariate analyses of factors associated with a six-month mortality.

**Parameters**	**Univariate analysis**		**Multivariate analysis**	
	**OR (95% CI)**	***P*** **-value**	**Adjusted OR (95% CI)**	***P*** **-value**
Age, years	0.99 (0.96–1.02)	0.521		
Male sex	1.06 (0.5–2.24)	0.880		
Cirrhosis	0.37 (0.11–1.2)	0.097		
Child-Pugh score	1.5 (0.96–2.35)	0.075		
Largest tumor size, cm	1.21 (1.12–1.3)	<0.001	1.09 (0.99–1.2)	0.072
Tumor number	1.13 (0.91-1.39)	0.277	1.31 (1.02-1.67)	0.038
TB, mg/dL	2.14 (1.24–3.7)	0.007		
AST, U/L	1.02 (1.01–1.03)	<0.001	1.04 (1.02–1.06)	<0.001
ALT, U/L	1.01 (1.00–1.02)	0.013	0.97 (0.95–0.99)	0.005
Albumin, g/dL	0.45 (0.23–0.91)	0.025		
Platelets, 10^9^/L	1.0054 (1.0025–1.0084)	<0.001		
AFP (≥400 *vs* < 400), ng/mL	4.79 (2.32–9.9)	<0.001	2.32 (0.95–5.66)	0.070

ABLI, albumin-bilirubin; AFP, alpha-fetoprotein; ALT, alanine aminotransferase; AST, aspartate aminotransferase; BCLC, Barcelona Clinic Liver Cancer; TB, total bilirubin.

### 3.3. Development of the model to predict a 6-month survival

Finally, five variables from the stepwise selection were selected and incorporated into the final model: tumor size, tumor number, AST, ALT, and AFP threshold of 400 ng/mL. Tumor size, tumor number, AST, and ALT levels were all encoded as continuous data, but AFP levels were encoded as categorical data (AFP <400 ng/mL vs. ≥400 ng/mL). The coefficients of variables derived from multivariate logistic regression analyses were multiplied by 100 to ease the calculation in the formula (Table 4). The final model developed in this study, namely FAIL-T (AFP, AST, tumor sIze, ALT, and Tumor number), was calculated using the following formula:

**Table 4:** The predictors and regression coefficients cooperating in the final model.

**Predictors**	**Regression coefficients**
AFP threshold of 400 ng/mL	0.839
AST (U/L)	0.036
Tumor size (cm)	0.088
ALT (U/L)	−0.027
Tumor number (n)	0.269

AFP, alpha-fetoprotein; ALT, alanine aminotransferase; AST, aspartate aminotransferase.

FAIL-T = (83.9 x AFP [<400 ng/mL:0, ≥400 ng/mL:1] + 3.6 x AST [U/L] + 8.8 x tumor sIze [cm] – 2.7 x ALT [U/L] + 26.9 x Tumor number)/50

The FAIL-T score ranged from 1 to 24.9 in the training set, and the AUROC of the model for predicting 6-month death was impressively high at 0.855 (95%CI: 0.801–0.911). The FAIL-T model was then validated in the validation set, and it still had an AUROC of 0.806 with scores ranging from −0.7 to 18.3.

### 3.4. The optimal cutoff value of the current model

As the purpose of the model is to identify patients who should not undergo TACE as their first-line treatment, the threshold for predicting death within 6 months was set at a high specificity (≥90% specificity to warrant that the patients would survive no longer than 6 months), and the trade-off of a low sensitivity was acceptable. An optimal threshold level of ≥8 met the aforementioned criteria. In the training and validation sets, patients with a FAIL-T score of ≥8 yielded a specificity of 94% and 90.7% for predicting 6-month mortality, respectively. At this threshold, the sensitivity was 50% and 52.4%, the NPV was 88.3% and 88.6, and the PPV was 67.7% and 57.9% for predicting the overall survival of less than 6 months in the training and validation sets, respectively.

### 3.5. Survival stratified by a FAIL-T score threshold of 8

**Figure 1:**
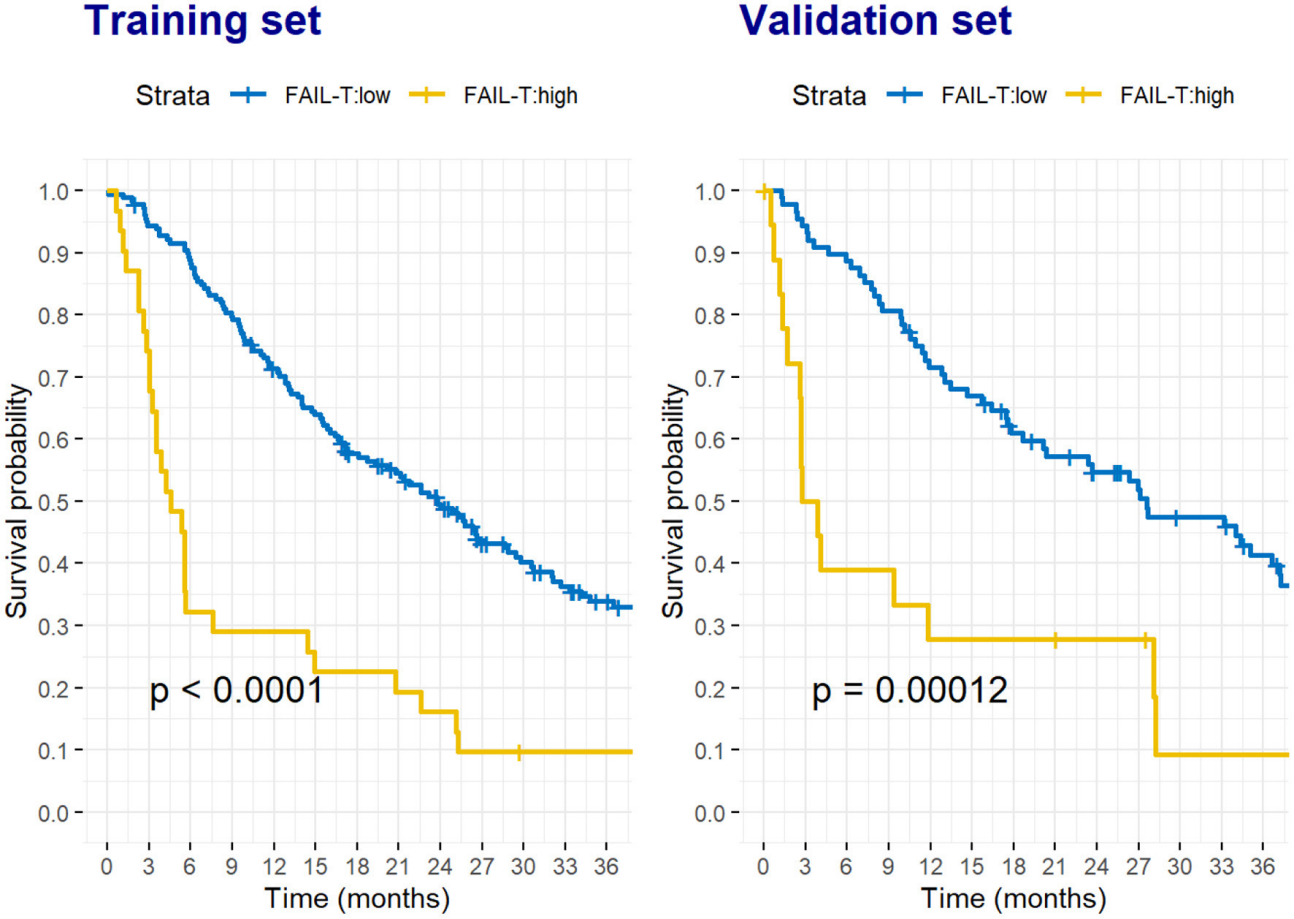
Survival probability of HCC patients stratified by the low and high FAIL-T scores in the training set **(A)** and validation set **(B)**. The formula of FAIL-T score was (83.9 x AFP [<400 ng/mL:0, ≥400 ng/mL:1] + 3.6 x AST [U/L] + 8.8 x tumor sIze [cm]−2.7 x ALT[U/L] + 26.9 x Tumor number)/50. A low FAIL-T score was defined as a score of <8, whereas a high FAIL-T score was defined as a score of ≥ 8.

When patients were stratified into high (≥8) and low (<8) FAIL-T groups, the differences in OS between the two groups were clear and consistent in both the training and validation sets (Figure 1).

In the training set, patients in the high FAIL-T group had a median OS of only 4.6 (95%CI: 3.2–14.4) months, while those in the low FAIL-T group had a median OS of 23.8 (95%CI: 18.6–29.4) months (*P* < 0.001). Similarly, in the validation set, the high FAIL-T group (3.3 [95%CI: 2.6–NA] months) had a significantly lower median OS than the low FAIL-T group (27.6 [95%CI: 20.1–37.2] months, *P* < 0.001).

After adjusting for age and sex in the Cox regression analyses, the FAIL-T score of ≥8 demonstrated a significantly increased instantaneous risk of death over time, with an adjusted hazard ratio (aHR) of 3.0 (95%CI: 1.96–4.5), and 3.0 (95%CI: 1.67–5.4) in the training and validation sets, respectively.

We also performed subgroup analyses based on the Child–Pugh score and HCC etiology. Those with FAIL-T scores ≥8 had significantly shorter OS in both the CTP-A5 group (Supplementary Figure 1A) and the CTP A6-B7 group, but the statistically significant level was not reached (Supplementary Figure 1B). Similarly, patients with FAIL-T score ≥8 had significantly lower OS in HBV HCC (Supplementary Figure 2A), but not in non-HBV HCC patients (Supplementary Figure 2B).

### 3.6. Comparison to the “six-and-twelve” score

The FAIL-T model was then compared to the “six-and-twelve” scoring system in order to predict a 6-month death, the AUROC of FAIL-T model was higher than the “six-and-twelve” scoring system in both the training and validation sets, with AUROCs of 0.855 vs. 0.750 (*P* = 0.001) and 0.805 vs. 0.728 (*P* = 0.107), respectively.

## 4. Discussions

In this study, we developed a prognostic model to predict patients with unresectable HCC BCLC up to B who would survive <6 months after their first TACE. These patients did not appear to benefit from TACE, though they may have experienced TACE-related complications. The FAIL-T score comprised five routine parameters in HCC patients, including the largest tumor size, the number of tumors, and liver function tests such as serum AST and ALT levels, and AFP level. All variables were routinely tested in HCC patients at baseline before any treatment decisions, allowing this scoring system to be easily applied in the general care of HCC patients. A high FAIL-T score (8 or higher) was significantly associated with poor 6-month survival. The score yielded a very good performance in both the training and validation sets (AUROCs of 0.855 and 0.805, respectively). With this newly developed score, TACE could be avoided in 14–18% of all patients with unresectable HCC BCLC stage A and HCC BCLC stage B, as they would not benefit from TACE treatment in terms of survival. Additionally, their chances of experiencing unnecessary post-TACE complications or adverse events may be reduced.

Tumor burden, as measured by its largest size and number of tumors, is a common factor that has been incorporated into many HCC prognostic models, similar to our scoring system (17, 28–31). Previous studies have shown that tumor burden is significantly associated with the survival rate of HCC patients (27, 32, 33). Unlike liver transplantation criteria (28, 34), there was no defined threshold for tumor burden to be an absolute contraindication for TACE. Although the “six-and-twelve” scoring system, which used only tumor burden parameters, could demonstrate differences in OS of patients in each score stratum (17), patients in the highest stratum still had a median survival of more than 6 months (16, 17). Therefore, its utility in helping physicians in determining which HCC patients are not suitable for TACE is limited. Moreover, subsequent external validation studies showed that the “six-and-twelve” score had a lower prognostic ability, with AUROCs ranging from 0.59 to 0.699 (16, 32, 35–37), when compared to the original Wang et al. (17) cohort (0.73).

In addition, the hepatic function should be preserved in patients undergoing TACE. Several studies found that patients with high AST and ALT levels have poorer survival (38–40). Moreover, AFP is a well-known tumor marker for both HCC diagnosis and prognosis. It has also been used in other HCC models (30, 31, 41–44). Bai et al. reported that AFP level correlated with a higher pathologic grade, more advanced stage, and shorter survival (45). Incorporating AST, ALT, and AFP levels, in addition to the tumor burden, into our model helps to reflect both tumor status (burden and biology) and hepatic function status. This might explain why the FAIL-T model was superior to the “six-and-twelve” scoring system in predicting 6-month survival in patients undergoing TACE.

It is widely known that the survival of patients with intermediate-stage HCC varies according to heterogeneity in tumor burden and patient characteristics. Bolondi's sub-classification (18) and the Kinki's criteria (19) were proposed earlier to facilitate treatment decisions in intermediate-stage HCC patients. However, these criteria had some limitations, particularly for the lack of survival data in different subclasses. Similarly, the recently updated 2022 BCLC system (4) recommended that patients with BCLC stage B may have more treatment options than TACE, as patients with infiltrative tumors and diffuse or extensive liver involvement may benefit from systemic therapy rather than TACE. Nonetheless, a consensus on when to justify diffuse or extensive disease and select treatment options other than TACE, as well as the survival data of those patients, are still needed in the updated recommendation. Our FAIL-T model might aid filling the gap in this scenario, as we demonstrated that intermediate-stage HCC patients with FAIL-T scores ≥8 mostly survived no longer than 6-month post-TACE. As such, other treatment options with a more favorable safety profile and better survival benefit should be considered in this group of patients. Furthermore, in countries with a high HCC burden, such as Asian countries, including Thailand, where healthcare facility resources are limited, removing those who are not good candidates for TACE from the list may shorten the waiting time and achieve a faster schedule for HCC patients who would benefit from TACE.

The strengths of our study are that we primarily developed a scoring system for naive HCC patients who then underwent TACE as their first and only treatment. Furthermore, we included patients with various etiologies of HCC, reflecting the real-world situation that physicians face when deciding which intermediate-stage HCC patients should undergo TACE. Moreover, our model comprised only routinely investigated laboratory and imaging data in most HCC patients: AFP, AST, ALT, the largest tumor size, and tumor number. Therefore, most physicians can calculate the score for HCC patients at no additional cost. The score showed a very good diagnostic performance, with AUROCs of over 0.80 in both the training and validation sets for predicting 6-month survival. In the current era of targeted therapy and immunotherapy for HCC, alternative treatment options might be considered for patients who do not benefit from TACE.

We acknowledge that the present study has some limitations. While the AUROC of FAIL-T outperformed the “six-and-twelve” scoring system in both data sets, the statistically significant level was not reached in the validation set, despite a substantial improvement in AUROC in the FAIL-T score (0.805) vs. “six-and-twelve” score (0.728). This finding might result from the small number of 107 patients in the validation cohort. Furthermore, because it was a single-center study, all patients were treated by two experienced interventional radiologists. Therefore, larger external validation of the FAIL-T score is needed to confirm the generalizability of the model. Additionally, all patients in our study underwent TACE without any other treatment options, and it is unknown whether other treatment modalities (e.g., systemic therapy) are more beneficial for those with FAIL-T ≥8, who did not respond well to TACE. Finally, because the study was conducted retrospectively, and we obtained the date of death from the Thailand Civil Registration database, we could not identify the cause of death precisely. It was not possible to determine whether the patients who survived <6 months died as a result of post-TACE complication, HCC progression, or other cause(s. We cannot ensure that the prognosis in this group of patients would have improved with systemic treatment. Therefore, further studies are required to determine the best treatment options for this group of HCC patients.

## 5. Conclusion

In patients with HCC, we developed and validated the FAIL-T score, which included routine imaging and laboratory parameters such as AST, ALT, AFP, tumor size, and tumor number. FAIL-T is a risk stratification tool that can identify HCC patients who are at high risk for 6-month mortality following their first TACE. TACE may not benefit patients with intermediate-stage HCC who have a high FAIL-T score (≥8). However, whether other systemic treatment options will be beneficial for those with FAIL-T ≥8 remains unknown, and further studies are required.

## Lay summary

Recently, an updated BCLC 2022 system has proposed treatment options for intermediate-stage HCC. However, there is no strict cutoff point for when not to choose TACE as the first-line treatment. Therefore, we developed and validated the “FAIL-T score” to help guide the selection of candidates for TACE in treatment-naive HCC patients. A high FAIL-T score, designated with a cutoff ≥8, is associated with a high 6-month mortality rate, and other treatment options should be considered. In terms of predicting 6-month survival, our scoring system outperformed the currently available “six-and-twelve” score.

## Data availability statement

The data that support the findings of this study are available on request from the corresponding author.

## Ethics statement

The studies involving human participants were reviewed and approved by Faculty of Medicine, Prince of Songkla University. Written informed consent for participation was not required for this study in accordance with the national legislation and the institutional requirements.

## Author contributions

AK and PS made substantial contributions to the study concept and design, data collection, data analysis and interpretation, and drafting of the manuscript. SA contributed to the data collection and drafting of the study protocol and manuscript. PW, SJ, NC, and TP made substantial contributions to the interpretation of the data and critical revision of the article. All authors contributed to the critical revisions and approved the final manuscript.
