# Supplementary material for: FAIL-T (AFP, AST, tumor sIze, ALT, and Tumor number): a model to predict intermediate-stage HCC patients who are not good candidates for TACE
Source: Front Med (Lausanne). 2023 May 2;10:1077842. doi: 10.3389/fmed.2023.1077842 (PMC10185803; doi:10.3389/fmed.2023.1077842)
Supplement: Supplementary file 1 [file Data_Sheet_1.PDF]

Figure legends

Supplementary Figure 1 The survival probability of HCC patients stratified by the low and high FAIL-T scores in the a) Child-Pugh score A5 and b) A6-B7

a) **CTP-A5**

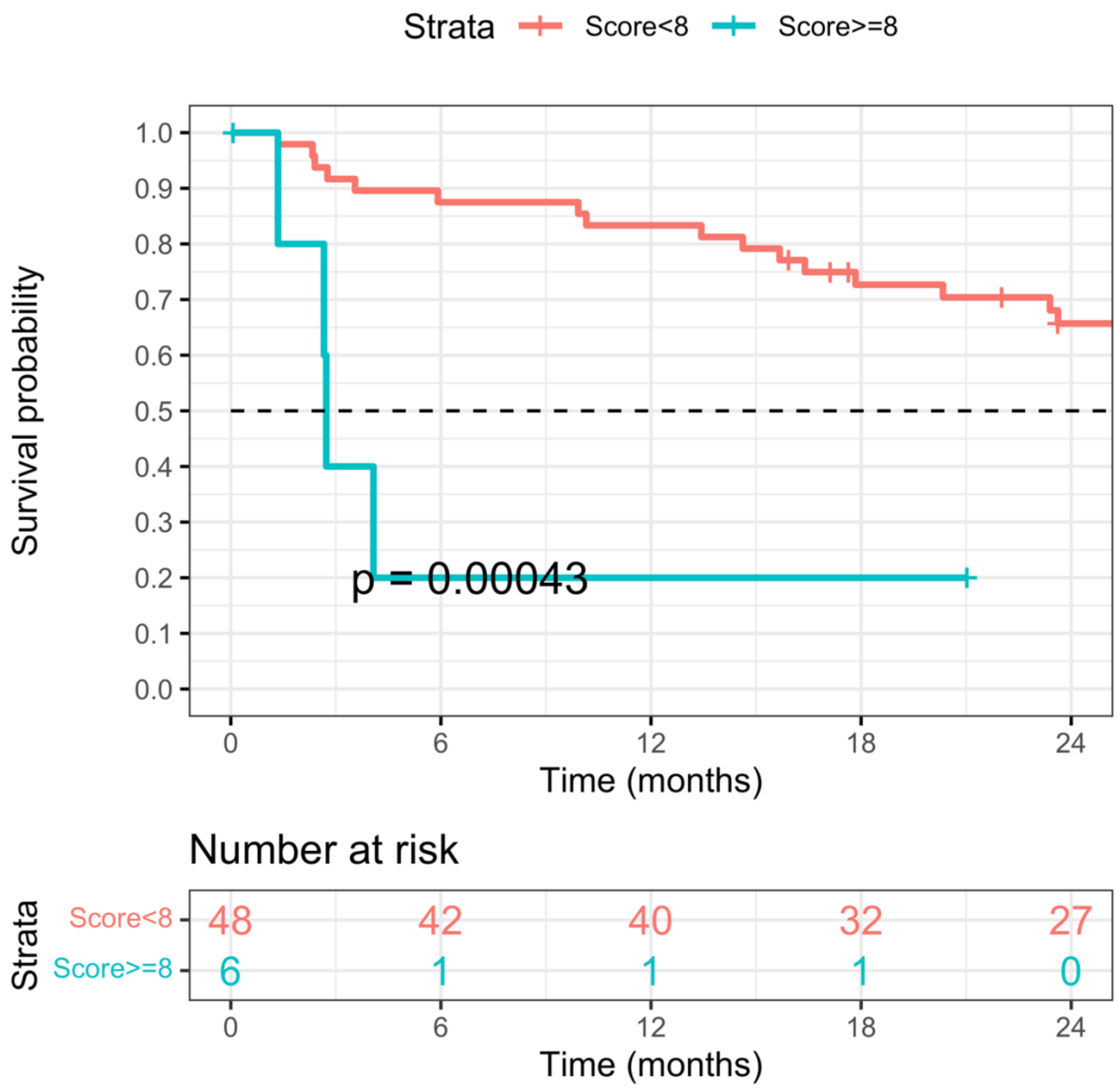

b) CTP A6-B7

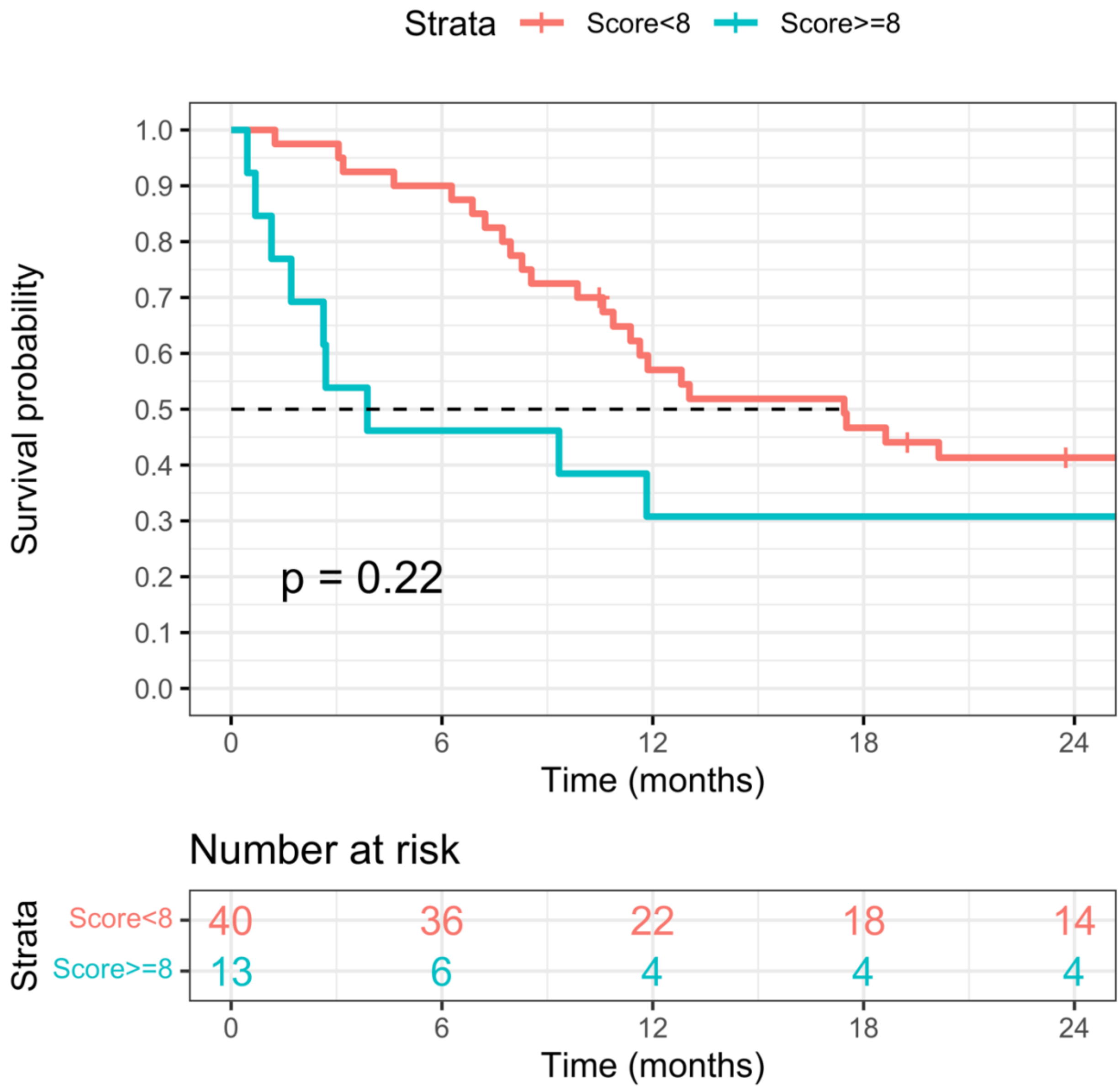

The formula of FAIL-T score was (83.9 x AFP [<400 ng/mL:0, >400 ng/mL:1] + 3.6 x AST [U/L] + 8.8 x tumor sIze [cm] – 2.7 x ALT[U/L] + 26.9 x Tumor number)/50. A low FAIL-T score was defined as a score of < 8, whereas a high FAIL-T score was defined as a score of ≥ 8.

Supplementary Figure 2 The survival probability of HCC patients stratified by the low and high FAIL-T scores in the a) HBV-HCC and b) non-HBV HCC

a) **HBV-related HCC**

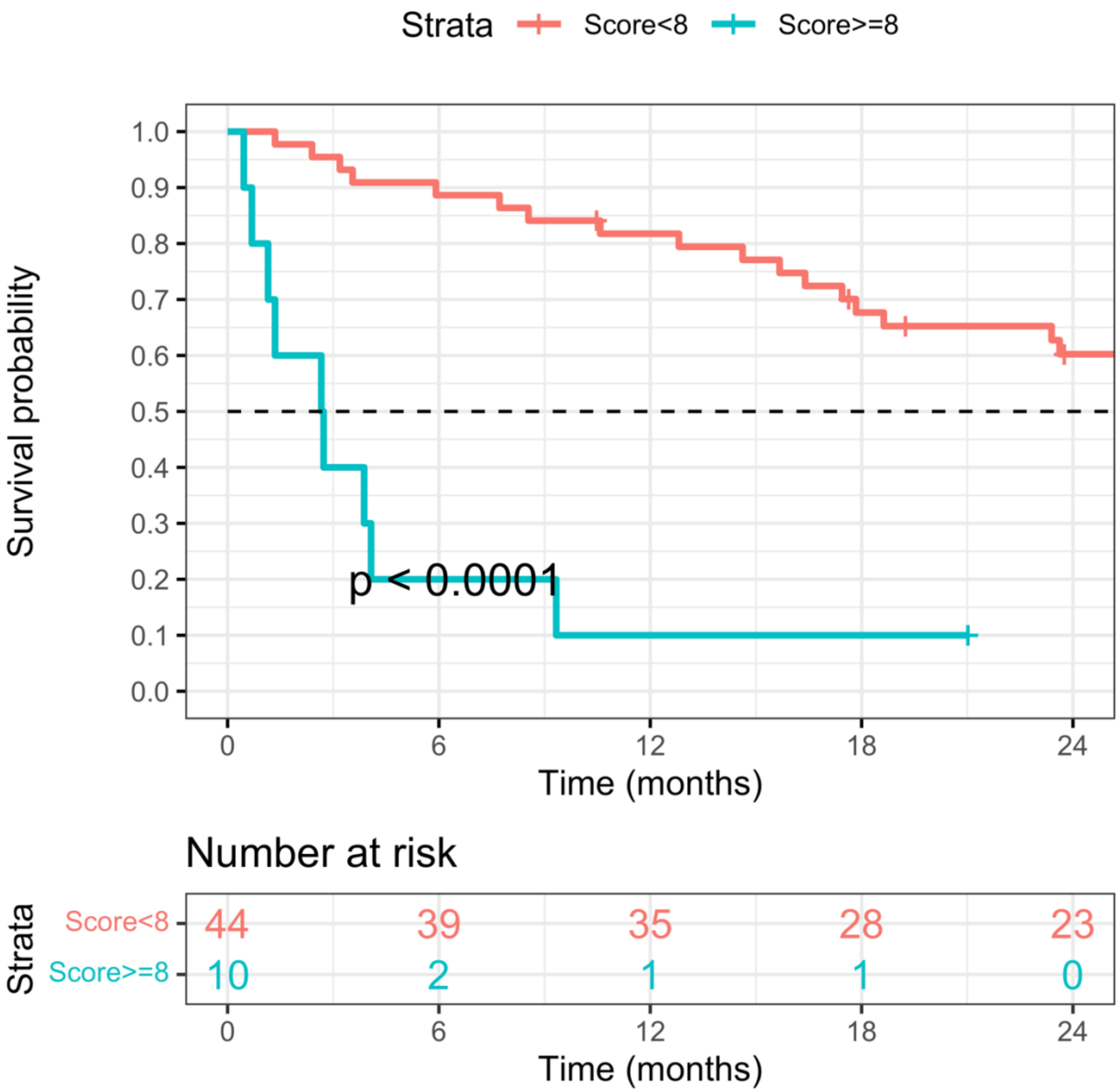

b) **Non-HBV related HCC**

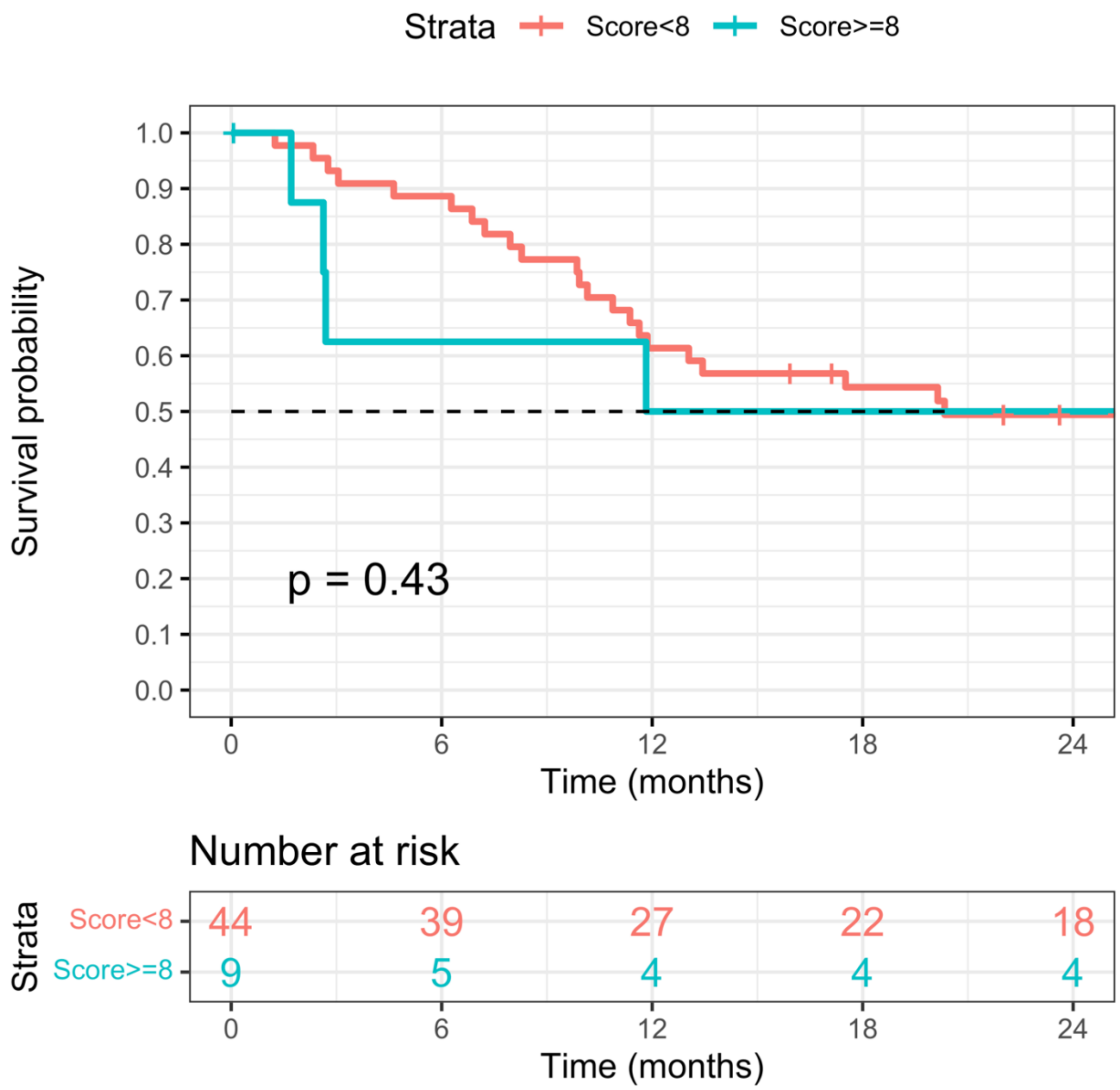

The formula of FAIL-T score was (83.9 x AFP [<400 ng/mL:0, >400 ng/mL:1] + 3.6 x AST [U/L] + 8.8 x tumor sIze [cm] – 2.7 x ALT[U/L] + 26.9 x Tumor number)/50. A low FAIL-T score was defined as a score of < 8, whereas a high FAIL-T score was defined as a score of ≥ 8.
